# Endometriosis and Adenomyosis: From Pathogenesis to Follow-Up

**DOI:** 10.3390/cimb47050298

**Published:** 2025-04-24

**Authors:** Francesco Giuseppe Martire, Eugenia Costantini, Claudia D’Abate, Giorgia Schettini, Giuseppe Sorrenti, Gabriele Centini, Errico Zupi, Lucia Lazzeri

**Affiliations:** 1 Department of Molecular and Developmental Medicine, Obstetrics and Gynecological Clinic, University of Siena, 53100 Siena, Italy; francescogmartire@libero.it (F.G.M.); eugenia.costantini22@gmail.com (E.C.); claudiadabate94@gmail.com (C.D.); giorgiaschettini@gmail.com (G.S.); centini.gabriele@gmail.com (G.C.); lucialazzeri79@gmail.com (L.L.); 2 Department of Gynecology, San Carlo Nancy Hospital, 00165 Rome, Italy; giuseppesorrenti@gmail.com

**Keywords:** adenomyosis, endometriosis, pathogenesis, management, symptoms

## Abstract

Endometriosis and adenomyosis are chronic, hormone-dependent disorders. Estrogens, in particular, play a pivotal role in the pathophysiology of these conditions. Understanding the disease mechanisms, including local hyperestrogenism and reduced progesterone sensitivity, is crucial for effective management. Early diagnosis is essential for appropriate therapeutic intervention, with medical hormonal treatment being the first-line approach. It is important to monitor patients over time and tailor hormone therapy to individual needs in order to optimize treatment adherence. Medical therapy not only enhances patients’ quality of life but also appears to slow disease progression in terms of both extent and severity. This narrative review aims to explore all aspects of endometriosis and adenomyosis, from pathogenesis to clinical symptoms, with particular emphasis on the role of hormones and the use of medical therapies.

## 1. Introduction

Endometriosis and adenomyosis are conditions of significant interest in the field of gynecology, both in terms of epidemiological relevance and due to their unclear etiopathogenesis and treatment-related challenges. These diseases are extensively studied by researchers, who contribute to an increasing number of hypotheses regarding their causative factors, as well as therapeutic approaches and correlations with comorbidities or physiological conditions, such as pregnancy.

Endometriosis and adenomyosis are chronic hormone-related diseases with an inflammatory basis. The prevalence of endometriosis is around 13–15% of the general female population of Western countries, rising to 30–40% of women with pelvic pain and up to 50–60% of women with infertility [1]. Symptoms, such as pelvic pain and infertility, impact the social life and quality of life of these young women. Endometriosis is often associated with adenomyosis, which can exacerbate symptoms and further impair fertility [2]. Hormones, particularly estrogens, play a crucial role in these pathologies [3]. Understanding the pathogenesis of the disease, particularly the local conditions of hyperestrogenism and reduced progesterone sensitivity, is crucial for effective disease management. It is essential to make an early diagnosis for adequate therapeutic management, in which medical hormone therapy is the first line of treatment. It is useful to follow these patients over time and adapt hormone therapy to individual patients to maximize compliance with therapy. In fact, the medical therapy, in addition to improving the patients’ quality of life, appears to reduce disease progression in terms of extent and degree [4]. Although many aspects of these conditions are well documented, a comprehensive framework that considers their frequent coexistence and overlapping symptomatology is still lacking. The aim of this narrative review is to provide an integrated analysis of endometriosis and adenomyosis, encompassing their pathogenesis, clinical manifestations, and diagnostic challenges. Furthermore, it proposes a dedicated follow-up and management approach tailored to affected patients, with the ultimate goal of improving their quality of life.

## 2. Materials and Methods

We performed an electronic literature search using the MEDLINE database to identify all English-language articles related to endometriosis, adenomyosis, and hormones published from the inception of the database through June 2024. We applied a combination of the following keywords and Medical Subject Headings (MeSH) terms to screen and select relevant studies: “Adenomyosis”, “Endometriosis”, “Estrogens”, “Hormones”, “Hormonal Factors”, “Hormonal Receptors”, “Progestins”, and “Therapy”. This review included a range of original research articles, such as randomized and non-randomized clinical trials, prospective observational studies, retrospective cohort studies, case-control studies, and review articles. Articles were considered eligible if they addressed the key topics of this narrative review, specifically providing insights into the relationship between endometriosis/adenomyosis and hormonal factors. The search and selection process was conducted independently by two researchers (F.G.M. and L.L.), who carefully reviewed all articles that met the inclusion criteria.

## 3. Prevalence

### 3.1. Endometriosis

Estimating the prevalence of endometriosis accurately is challenging. It is believed that there is a delay of at least 5–8 years between the onset of the first symptoms and the diagnosis, which impacts prevalence estimates [5,6]. Until recently, easy access to surgery and the compilation of epidemiological data were mostly available in industrialized countries with predominantly Caucasian populations. One of the earliest studies, conducted in Rochester, Minnesota, found that the annual incidence confirmed by surgical or histological diagnosis was 0.3% for women aged 15 to 49 years [7]. Other studies, carried out on selected populations, reported an annual incidence between 0.1% and 0.3% [8,9]. However, these figures are likely influenced by the tendency of both healthcare providers and patients, often due to cultural factors, to underestimate symptoms such as dysmenorrhea and chronic pelvic pain, which can serve as warning signs. Additionally, the diagnosis of this condition is often solely based on surgical intervention.

Currently, non-invasive diagnostic methods like transvaginal ultrasound (TVUS) and Magnetic Resonance Imaging (MRI), performed by skilled practitioners, are highly effective in diagnosing endometriosis without requiring histopathological confirmation [1]. According to more recent data, the prevalence of endometriosis is estimated to be around 3–5% in the general female population of reproductive age. However, this figure rises to 30% in infertile women and 50% in those with pelvic pain [10,11,12]. Moreover, in adolescent girls with pelvic pain unresponsive to medical treatment, the prevalence can reach 65–70% [13,14]. Research has also shown that women with early menarche, polymenorrhea, pelvic pain, infertility, and nulliparity are at greater risk of developing endometriosis [15]. Furthermore, endometriosis has been diagnosed in 50% of adolescent girls reporting severe dysmenorrhea [13].

### 3.2. Adenomyosis

The exact prevalence of adenomyosis remains unclear, as most estimates rely solely on diagnoses made after surgery, which likely leads to an underestimation of the true occurrence of the condition [16]. In patients undergoing hysterectomy, the estimated prevalence of adenomyosis over the past five decades has varied between 8.8% and 61.5% [17]. This broad range is often attributed to the lack of standardized histopathologic diagnostic criteria, variations in the number of tissue samples analyzed, and differences in the expertise of pathologists evaluating the specimens. It is estimated that at least nine different histopathological criteria are used for diagnosing adenomyosis [18]. Bergholt et al. (2001) [19] reported that the prevalence of adenomyosis increased to 18% when myometrial hyperplasia was absent. The prevalence rose significantly (from 31% to 61.5%) when three blocks of the uterine wall or six blocks of extra-uterine tissue were analyzed in histopathologic examinations. Based on these observations, Bird et al. (1972) [20] suggested that nearly half of adenomyosis cases in hysterectomy patients went undiagnosed when relying on less stringent diagnostic criteria, which required a minimum distance of 5 mm between the endometrium and the junction, along with the presence of myometrial hyperplasia. However, using more lenient criteria, such as a depth of ≥1 mm, increased the detection rate.

Prevalence estimates based solely on histopathological diagnoses have contributed to the perception that adenomyosis is primarily a condition affecting older women.

The first study to assess the prevalence of adenomyosis using ultrasound was conducted in the UK, involving 985 women who underwent TVUS for various conditions, including menorrhagia, pelvic pain, infertility, menstrual irregularities, amenorrhea, or abnormal postmenopausal bleeding. This study found a prevalence of 20.9% for adenomyosis [21]. A subsequent study focused on premenopausal women with regular menstruation in the previous 60 days, reporting a prevalence of 21.9% [22]. A similar study in Italy, which included 156 nulliparous women aged 18 to 30 years, found that 34% had adenomyosis [23]. The inclusion criteria for this study were regular menstrual cycles, no use of estrogen–progestin therapy, no history of infertility, and no ultrasound evidence of endometriosis or leiomyomas. These findings suggest that adenomyosis may develop earlier than previously believed based on histopathological data. Similar to histologic diagnosis, there is no universal consensus on the imaging criteria needed to diagnose adenomyosis, which can affect prevalence estimates. While ultrasound diagnosis is influenced by the operator’s experience and concurrent hormonal treatments [24], it remains a highly effective tool for detecting adenomyosis [16]. Both TVUS and MRI demonstrate reasonable effectiveness in diagnosing the condition. A recent study found sensitivities of 78%, 74%, and 84% for MRI, 2D TVUS, and 3D TVUS, respectively, with specificities of 88%, 76%, and 84% [24].

Adenomyosis is more commonly observed in women with endometriosis and infertility, with prevalence rates ranging from 35% to 79% [25,26,27]. It is also prevalent in women with both endometriosis and pelvic pain, with prevalence estimates ranging from 38% to 87% [28,29]. Among patients with deep infiltrating endometriosis, the prevalence of adenomyosis remains similarly high, ranging from 35% to 78% [30,31,32]. Notably, the highest prevalence has been reported for the focal adenomyosis of the external myometrium, with figures ranging from 49% to 97% [30,33,34].

## 4. Pathogenesis

### 4.1. Endometriosis

**Figure 1:**
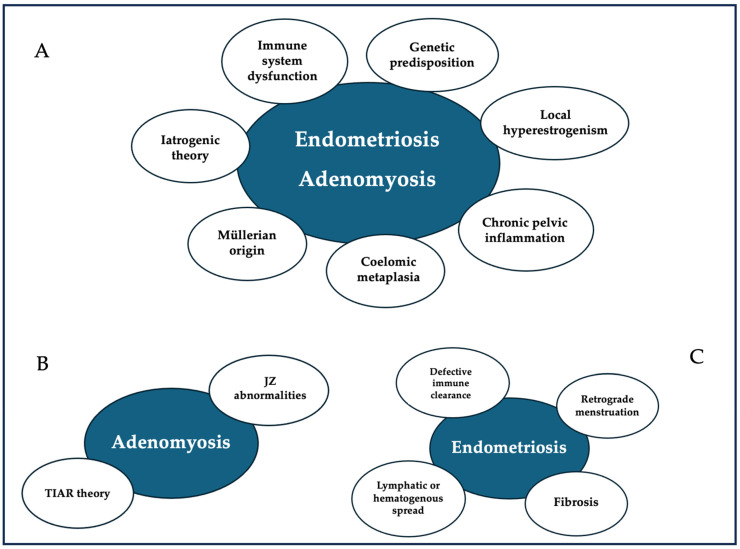
Pathogenesis theories: (**A**) common pathogenetic factors of endometriosis and adenomyosis; (**B**) specific pathogenetic factors of adenomyosis; (**C**) specific pathogenetic factors of endometriosis.

The main question, still unresolved, certainly concerns the origin of the endometrial glands and stroma in the ectopic site. In this regard, various hypotheses have been put forward since 1870, particularly following Von Rokitansky’s first description of the pathology in the mid-19th century [35]. Some hypotheses retain a mere historical interest, others are considered plausible to this day. In any case, none of these is able to fully define the pathogenesis of endometriosis, nor the different presentations of the disease [3]. Having said this, the most corroborated theses will be reviewed below (Figure 1).

#### 4.1.1. Sampson’s Theory: Retrograde Menstruation

The theory of retrograde menstruation remains one of the most widely accepted explanations for the pathogenesis of endometriosis, as it accounts for many of the disease’s observed features. Initially proposed by Sampson, this hypothesis suggests that during menstruation, viable endometrial cells, along with cytokines and growth factors, may reflux through the fallopian tubes into the peritoneal cavity, where they can adhere to surrounding tissues and proliferate under hormonal stimulation. This concept is supported by the histopathological resemblance between ectopic and eutopic endometrial tissue and by the frequent localization of lesions within pelvic organs. The anatomical positioning of the sigmoid colon may facilitate the retention of refluxed endometrial fragments, creating a microenvironment conducive to implantation.

Experimental models have further substantiated this theory; for example, a 2009 study demonstrated that surgical induction of retrograde menstruation in non-human primates resulted in endometriotic lesion formation in 50% of cases [36]. Nonetheless, retrograde menstrual debris is observed in up to 90% of menstruating women, yet only a minority—approximately 10%—develop the disease. This disparity suggests additional contributing factors, including excessive menstrual flow, intrinsic abnormalities of the eutopic endometrium, an impaired immune response, or an altered peritoneal milieu promoting angiogenesis and immune evasion.

Moreover, Sampson’s hypothesis has evolved to incorporate the role of endometrial stem/progenitor cells, which have been isolated from menstrual and peritoneal fluid. These cells, potentially shed from either eutopic endometrium or preexisting superficial lesions, may persist and differentiate under favorable conditions. Genetic predisposition, somatic mutations, or epigenetic modifications may endow these progenitor cells with a survival and proliferative advantage, enhancing their capacity to initiate lesions. While this model adequately explains pelvic and abdominal manifestations, it falls short in accounting for extra-pelvic disease spread [37].

#### 4.1.2. Coelomic Metaplasia Theory

In response to the limitations of the retrograde menstruation theory, in 1942, Gruenwald supported the thesis that mesothelial cells of any organ, including those of the pelvic cavity—the ovary in particular—can undergo differentiation into functional endometrium [38]. Endometriotic tissue would develop as a result of metaplasia of the peritoneal serosa by in situ transformation, whereas, according to the Müllerian remnant theory, cells present at Müller duct remnants would retain the ability to differentiate into endometriotic tissue following hormonal stimuli.

This hypothesis is corroborated, firstly, by the occasional occurrence of endometriosis in amenorrhoeic women and men undergoing hormone therapy; secondly, by findings of lesions in the pleural cavity, diaphragm, brain, and various other organs where the mesothelium is present [38].

#### 4.1.3. Benign Metastasis Theory

Of lesser relevance is the hypothesis of the spread of endometriotic metastases, either from the eutopic endometrium or from a focus of endometriosis, by dissemination via the bloodstream—in particular through the venous system—and by lymphatic dissemination. The most important evidence in support of this thesis comes from histological findings showing lesions in extra-pelvic sites, such as bone, lungs, and encephalon [39].

#### 4.1.4. Role of Fibrosis

In recent years, fibrosis has emerged as a key player in the etiopathogenesis of endometriosis. Rather than being merely a consequence of stromal cell transdifferentiation into smooth muscle-like cells, fibrosis may result primarily from the local tissue response to ectopic endometrial implants. This fibrotic reaction contributes significantly to disease progression and symptomatology. Fibrosis underlies many of the clinical manifestations of endometriosis, including the formation of adhesions and chronic pelvic pain. It represents a pathological process that not only reflects tissue remodeling but also perpetuates itself over time. The fibrotic milieu may promote further ectopic tissue survival and lesion stability [40].

Thus, fibrosis is not just a byproduct but a driving force in the chronicity of the disease. It plays a central role in lesion consolidation and resistance to therapy. Furthermore, its presence correlates with disease severity and poor surgical outcomes. Recognizing fibrosis as a self-reinforcing pathological hallmark opens new avenues for therapeutic intervention [41].

#### 4.1.5. Iatrogenic Theory

This hypothesis would explain the presence of endometriosis lesions on laparotomic scars from operations on the pelvic organs or at the vulvo-perineal level. More rarely, formations are seen on caesarean section scars or on sutures from lacerations in spontaneous deliveries.

#### 4.1.6. Immune System Abnormality Theory

The altered immunological response in patients with endometriosis has long been known to be impaired; it has been suggested that a deficiency in immunosurveillance may diminish the ability to eliminate refluxed menstrual debris, thereby promoting the persistence of ectopic endometrial cells within the pelvis and promoting their proliferation [38]. In peritoneal fluid, an increased concentration of macrophages, i.e., cells of the immune system that are responsible for recognizing foreign or damaged cells, which, once detected, are processed and presented to T lymphocytes. However, in patients with immune malfunction, macrophages are induced to secrete growth factors and cytokines, stimulating the survival of ectopic cells. Similarly, altered cytokine production causes a change in peritoneal fluid, thus contributing to an environment favorable to the proliferation of ectopic endometrial tissue. The regulation and activation of macrophages and lymphocytes is finely controlled by cytokine expression, resulting in a particularly delicate balance that endometriosis inevitably alters [42,43].

#### 4.1.7. Genetic Predisposition Theory

A hereditary component has also been recognized in endometriosis: the involvement of genetic factors in its development is supported by various research studies. In particular, a study carried out in Australia on 3096 twins concluded that approximately 51% of the variance in latent predisposition to endometriosis was attributable to additional genetic influences [44]. Further analyses were performed on sets of twins recruited by the American Endometriosis Association and the United Kingdom Endometriosis Society and found the concomitant presence of endometriosis in 14 out of 16 sets of twins, including nine sets with moderate-to-severe lesions [45]. Furthermore, in women with first-degree relatives with endometriosis, the risk of developing the disease increases by 5–7% [46]. To date, many deregulated genes have been identified in endometriosis cells with a wide variety of functions, including apoptosis, cell cycle regulation, vascularization, immune regulation, and cell adhesion [47,48].

The most recent hypotheses seek answers in the field of epigenetics. The latter concerns heritable changes in gene expression that can be influenced by environmental factors and are not the result of changes in the DNA code. Epigenetic mechanisms, including DNA methylation, imprinting loss, and gene regulation by microRNAs, can influence the growth and invasiveness of endometrial cells, as well as the inflammatory response, all of which are crucial factors in the development and progression of endometriosis. Understanding these epigenetic changes could pave the way for new therapeutic strategies to treat the disease. Epigenetics has revolutionized the understanding of many complex multifactorial diseases such as cancer [49,50] and, recently, the possibility has emerged that epigenetic mechanisms probably play a significant role in the origin and progression of endometriosis [38,51].

#### 4.1.8. The Role of Hormones

The reason why transplanted or congenital ectopic endometrium develops into endometriosis is the source of much research. The manifestation of the pathology in the reproductive age and its regression after menopause or after ovariectomy [52] suggests a key role of ovarian steroid hormones. Current investigations focus on the role of estrogens and their receptors, estrogen-dependent physiological and molecular changes [53], local levels of these hormones [53,54], and intracellular production linked to aromatase activity.

The local production of these hormones and the loss of protective mechanisms result in higher estradiol levels found in both eutopic and ectopic endometria in patients with endometriosis compared to unaffected women [55]. In addition, increased estrogen production results in a positive feedback loop, further increasing estrogen production through induction of the enzyme cyclooxygenase type 2 (COX-2); subsequently, elevated levels of prostaglandin E2 further stimulate aromatase activity [56,57].

It is interesting to note that local estrogen production is the result of the activation of microtrauma-induced tissue injury and repair (TIAR) mechanisms in the basal endometrial layer [3]. The basal layer exhibits characteristics typical of stem cells and is distinguished by its capacity for dislocation and proliferation, properties that are enhanced in women with endometriosis [58,59]. Ectopic basal endometrial debris in the peritoneal cavity can induce chronic local inflammation and activate TIAR mechanisms, promoting a cascade reaction characterized by local estrogen production, proliferation, and infiltrative growth, resulting in endometriotic lesion formation [60,61]. The microenvironment that is created is capable of activating peritoneal macrophages with subsequent secretion of pro-inflammatory cytokines, such as tumor necrosis factor α (TNF-α) and interluchin-1β (IL-1β), which stimulate the activation of nuclear factor kβ (NF-kβ). In addition, the expression of endothelial growth factor (VEGF) is induced, as well as the activation of the cell cycle and the anti-apoptotic gene Bcl-2 [62,63].

The synergistic counterpart of estrogen overproduction, together with overexpression of its receptors, is identified in progesterone resistance in endometriotic tissues, which prevents the modulation of genes involved in decidualization, cell cycle regulation, and the estrogen inhibitory response [64]. Progesterone resistance is particularly present in the ectopic endometrium compared to the eutopic endometrium, although it has also been identified in the eutopic endometrium of women with endometriosis compared to control cases [65].

The main mechanism responsible for resistance is the under-regulation of progesterone receptors in ectopic tissue, which results in variation in the expression of target genes, such as the gene encoding 17β-hydroxysteroid dehydrogenase (17β-HSD) [64,66].

The pathways potentially underlying progestin suppression are multiple, e.g., the concentration of pro-inflammatory cytokines, such as TNF-α and IL-1β, which are involved in chronic inflammation, and the TIAR mechanism, which is reported to be directly related to progesterone expression [67]. It is, therefore, possible to argue that altered hormone expression plays a fundamental role in the pathogenesis and evolution of endometriosis.

The developmental process of endometriosis is variable and may occur through the progressive acquisition of alterations in the physiological processes of the endometrium, including altered hormonal physiology and modulating the interaction between endometriotic lesions and the inflammatory response. However, the heterogeneity of endometriosis and the different contexts in which it develops suggest that a single model of etiopathogenetic explanation is not satisfactory [3].

### 4.2. Adenomyosis

The etiopathogenesis of adenomyosis, although not completely defined, is complex and involves several biological factors, including hormonal, inflammatory, and genetic alterations (Figure 1).

#### 4.2.1. The Role of Hormones

Hormonal regulation is one of the key aspects in the pathogenesis of adenomyosis. Estrogens are known to promote the proliferation of endometrial tissue. In adenomyosis, an altered hormonal microenvironment is believed to promote the invasion of endometrial tissue into the myometrium. Increased estrogen production can lead to excessive enlargement of endometrial tissue, which is more likely to infiltrate the uterine muscle. Women with adenomyosis show increased expression of estrogen receptors (ERs) in the myometrium and endometrium, suggesting a hypersensitive response to estrogen. This increase in receptors could favor the growth and invasion of endometrial tissue within the uterine wall [4]. In addition, increased activity of 5-alpha-reductase is observed in the myometrium, an enzyme that converts testosterone to dihydrotestosterone (DHT), which has an estrogen-like effect in promoting endometrial tissue growth [68]. Another crucial aspect concerns estrogen metabolism. In many women with adenomyosis, there is a dysregulation in estrogen metabolism that can lead to elevated levels of unmetabolized estrogens, such as estrone (E1), compared to estradiol (E2). An altered balance between these estrogens can promote the growth of endometrial tissue within the myometrium [69].

Progesterone resistance is another key mechanism in adenomyosis. Women with adenomyosis often have reduced progesterone receptor (PR) expression in the myometrium, as well as dysfunction in progesterone signaling pathways. This leads to a reduced response to progesterone, which would normally inhibit the growth of endometrial tissue and promote its maturation and stability [70]. Progesterone resistance could be a contributing factor to the excessive proliferation of endometrial tissue in the myometrium.

#### 4.2.2. Immune System Abnormality Theory

Besides hormones, inflammation plays an important role in the etiopathogenesis of adenomyosis. The uterine inflammatory microenvironment can be favored by the hormones themselves, but also by genetic and environmental factors. Chronic inflammation promotes angiogenesis that facilitates the invasion of endometrial tissue into the myometrium. Pro-inflammatory cytokines such as TNF-α, IL-6, and others may be elevated in women with adenomyosis and stimulate endometrial tissue proliferation [71].

#### 4.2.3. Genetic Predisposition Theory

Recent studies suggest that adenomyosis may be influenced by a genetic predisposition. Women with a family history of adenomyosis or other gynecological conditions, such as endometriosis, have an increased risk of developing the disease. Mutations in genes that regulate cell proliferation, such as KRAS and PIK3CA [4], and differentiation could increase the likelihood of endometrial tissue invading the myometrium [68].

Furthermore, epigenetics, i.e., changes in gene expression that do not involve mutations in DNA, may be implicated in adenomyosis. Epigenetic modifications, such as DNA methylation and post-translational modifications of proteins, may alter the activity of genes that regulate hormonal response and endometrial tissue growth [70].

However, in recent decades, new and more complex theories have emerged that integrate hormonal, epigenetic, genetic, and inflammatory components. The epigenetic theory, in particular, has attracted great interest as it seems to provide an explanation for the persistence and abnormal proliferation of endometrial tissue in the myometrium. In this context, hormonal mechanisms, receptors for estrogen and progesterone, and advanced theories such as TIAR and coelomic epithelium have become crucial topics of discussion.

Epigenetics refers to changes in gene expression that do not involve DNA mutations but alter the regulation of the genes themselves. These changes can be influenced by environmental, hormonal, and inflammatory factors. In the context of adenomyosis, epigenetic alterations play an important role in interfering with the regulatory mechanisms of cell proliferation and differentiation of endometrial tissue.

Recent studies have shown that women with adenomyosis have altered DNA methylation, particularly in genes that regulate the growth and invasiveness of endometrial tissue. DNA methylation is a process that can suppress or activate gene expression, and alterations in this process could contribute to an aberrant response to hormonal stimuli [72].

Another epigenetic mechanism involved is the modification and acetylation of histones, which may facilitate or inhibit gene transcription. These epigenetic changes could alter the endometrium’s response to estrogen and progesterone, crucial factors in the control of the menstrual cycle and endometrial tissue growth [69].

#### 4.2.4. The Tissue Invasion and Repair Hypothesis (TIAR)

TIAR proposes that adenomyosis results from a distortion of the tissue repair process that occurs after menstruation. Specifically, during the menstrual cycle, retrograde menstrual flow may damage the myometrium and stimulate a reparative response that, in the presence of abnormal growth factors or alterations in hormonal control, leads to invasion of the myometrium by endometrial tissue.

This theory suggests that endometrial epithelium, instead of being completely expelled, may infiltrate the myometrium due to an alteration in tissue repair mechanisms and regulation of the inflammatory response. Alterations in the uterine microenvironment and excessive stimulation by estrogen may favor the survival and proliferation of infiltrating endometrial cells [73].

#### 4.2.5. Coelomic Epithelium Theory

Another advanced mechanism in the etiopathogenesis of adenomyosis is related to the coelomic epithelium theory, which is based on the idea that endometrial epithelium cells may result from aberrant differentiation of coelomic mesothelial cells. According to this theory, endometrial tissue would not only originate from retrograde infiltration of menstrual flow but could also result from a transformation of coelomic mesothelial tissue into endometrial tissue within the myometrium.

This hypothesis suggests that, under the influence of hormonal factors, mesothelial cells that normally line the peritoneal cavity may acquire endometrial characteristics and penetrate the myometrium. The ability of these cells to respond to hormonal stimuli could underlie endometrial proliferation and invasion in women with adenomyosis [74].

Recently, the hypothesis has been put forward that Schwann cells (SCs) may play a role in the development of adenomyosic lesions. The border region between myometrium and endometrium (EMI) has endometrial stem cells and is densely innervated by peripheral nerves lined with SCs [75,76]. Indeed, an adenomyosic lesion could result from dedifferentiated Schwann cells (dSCs). Following injury, SCs transform into a non-myelinated state [77], causing changes in EMI, mediated by the overexpression of several genes involved in tissue repair [78]. These changes are collectively known as myometrial interface dysfunction (EMID). A theory has been formulated about the central role of SDCs in this process, suggesting that dedifferentiated Schwann cells can differentiate into endometrial epithelial cells under the influence of multiple stimuli, including growth factors, estrogen, and inflammatory cytokines [2,79,80].

## 5. Symptoms

### 5.1. Endometriosis

Women with endometriosis face a considerably higher risk of infertility than the general female population, with estimates ranging between 10% and 30% [81,82,83].

In addition to the more common symptoms, less frequently reported symptoms include fatigue, low mood, bloating, frequent urination, and sleep disturbances, which are often associated with the ongoing pain [5,84,85,86].

Although rare, extrapelvic symptoms such as shoulder pain, catamenial pneumothorax, or cyclic sciatica may occur when endometriosis affects atypical sites, including the lungs or sciatic nerve [87].

For adolescent girls, the impact of painful symptoms is often overlooked, even though these symptoms significantly affect their daily activities and overall quality of life. As a result, diagnosis is often delayed compared to adults [88,89]. Additionally, adolescents tend to experience symptoms like early-onset dysmenorrhea, nausea, gastrointestinal issues, and non-cyclic pain more frequently [4,90,91,92].

### 5.2. Correlation Between Different Features of Endometriosis and Symptoms

Endometriosis-related symptoms can considerably affect the general health and mental and social well-being of affected women. There are currently no studies demonstrating a relationship between the extent of the disease and its symptomatology [93]. However, there appears to be a strong correlation between symptoms and the location of endometriosis lesions (Table 1). Studies show that women with deep infiltrating endometriosis (DIE) may have dysfunction of the pelvic floor and lower limb muscles, and painful symptoms associated with hypertonia and difficulty relaxing the pelvic muscles [94]. It is also worth noting that patients with lateral parametric endometriosis report an increased frequency of constipation and symptoms of impaired urination [95,96]. Following a retrospective study, which included 225 patients with pelvic pain and DIE, a correlation was found between the frequency of severe dysmenorrhea and the presence of adhesions in Douglas’ cord. Dyspareunia is, on the other hand, typically associated with involvement of the uterosacral ligaments, the frequency of chronic non-cyclic pelvic pain appears to be higher when DIE involves the bowel, while dyschezia is more frequent when the vagina is infiltrated [97,98]. Within the framework of the clinical manifestations of endometriosis, the spectrum of infertility, understood as the inability to conceive after a year of regular, unprotected intercourse, is predominant. In fact, the latter affects 30–50% of women with endometriosis. Among them, the monthly pregnancy rate is only 2–10% compared to 15–20% in the healthy population [99].

Despite the clinically recognized association between endometriosis and infertility, the mechanisms implicated in endometriosis-associated infertility are unclear, and this condition is currently considered multifactorial. The role of pain in the spectrum of infertility is not always taken into account, and there are few studies on the subject; however, superficial and deep dyspareunia and chronic pelvic pain should not be underestimated in a woman’s sexual sphere. Endometriosis is associated with a ninefold increased risk of deep dyspareunia mostly due to the infiltrative form and severe stages of the disease affecting the posterior vaginal fornix, the pouch of Douglas, the uterosacral ligaments, and the rectum [100,101,102]. Chronic, no menstrual pelvic pain associated with the disease might influence sexual life by reducing desire, frequency of sexual intercourse, arousal, or orgasm [103,104].

Among the main causes of infertility in patients with endometriosis are certainly pelvic adhesions, which are typical in affected patients, both due to the inflammatory nature of the disease and any previous surgery. Adhesions can hinder the movement of the fallopian tubes, preventing the capture of the oocyte released during ovulation. Moreover, it is well established that endometriomas compromise the quality of ovarian tissue, leading to alterations in follicular and vascular patterns [105].

Not to be forgotten is the altered pelvic anatomy due to synechiae, thus reducing the possibility of natural fertilization as the normal positioning of the reproductive organs is compromised [106,107].

Endometriosis and obstetric complications appear to share common pathophysiological mechanisms, including abnormal activation of inflammation, structural and functional changes in the junctional zone, and changes in uterine contractility [108].

The pathology under investigation may be associated with alterations in ovulation and oocyte production, increased inflammatory cells in the peritoneal fluid, and ovarian endometrioma, all of which may compromise normal embryonic development. It is possible for such disorders in the peri-implantation period to be perpetuated during the later stages of pregnancy, resulting in adverse outcomes for the mother and fetus [109].

Specifically, endometriosis induces changes in the expression of local and systemic cytokines that alter the primary function of the endometrium. Within the latter, there is an induction of the expression of p450 aromatase, a cytochrome that causes a shift from progesterone to estrogen activity and promotes the growth and development of endometriosis. Furthermore, inflammation is known to influence the expression of steroid receptors and aromatase. Peritoneal fluid in affected patients is rich in interleukin-17, stimulating Cox-2 activity and the expression of interleukin-6, interleukin-8, and aromatases. Estrogen receptor 1 (ER1) is usually downregulated in the secretory phase at the time of implantation.

However, in endometriosis, this phenomenon is deficient, and implantation failure may occur frequently. According to the literature, the implantation rate in the group of endometriosis is 1.5 times lower than infertile patients without endometriosis [110].

The switch to estrogen dominance generates factors that promote immunosuppression, angiogenesis, inflammation, and cell proliferation. Under physiological conditions, progesterone triggers a response in the endometrium that induces and prolongs implantation of the embryo, called decidualization. The decidua is of extreme importance, being the mother–embryo interface. It protects the embryo from immune rejection, provides nutrients, and modulates trophoblast invasion. Under physiological conditions, progesterone plays a key role in modulating the endometrial environment by suppressing inflammation and reducing uterine contractility. In the context of endometriosis, however, a state of progesterone resistance is frequently observed, contributing significantly to impaired embryo implantation through the promotion of a pro-inflammatory milieu. This disease is characterized by a microenvironment rich in inflammatory mediators, angiogenic factors, and hormonal activity, all of which may negatively influence reproductive outcomes. Several mechanisms have been proposed to explain implantation failure in affected individuals, including oocyte dysfunction, compromised embryo quality, hormonal dysregulation, diminished expression of endometrial receptivity markers, and insensitivity to progesterone signaling [111].

### 5.3. Adenomyosis

Adenomyosis can greatly affect women’s quality of life, manifesting itself in signs such as menstrual pain, heavy menstrual bleeding, and possible difficulties with fertility. However, about one-third of those affected have no obvious symptoms. It is essential to identify the signs that could indicate the presence of adenomyosis [80,112].

One of the most common symptoms of adenomyosis is chronic pelvic pain, often described as a feeling of heaviness or a dull ache, located in the lower abdomen or pelvic area. It occurs most intensely during the menstrual period and may be accompanied by cramp-like pain. The pain is related to inflammation and increased size of the uterus caused by the presence of endometrial tissue in the myometrium [113]. Persistent inflammation in the uterus can irritate pelvic nerves, increasing sensitivity and contributing to chronic pain [114].

Dysmenorrhea is a classic symptom of adenomyosis and can be classified into primary and secondary. Primary dysmenorrhea is functional pain without an organic cause, typically mild or moderate. Secondary dysmenorrhea indicates an underlying condition, often related to endometriosis or adenomyosis, and is usually more severe [115].

Patients with adenomyosis frequently and significantly present with heavy menstrual bleeding or menorrhagia. The presence of endometrial tissue in the myometrium causes a dysfunctional menstrual cycle, resulting in endometrial hyperplasia and profuse bleeding. Bleeding can be so profuse as to cause secondary anemia in patients, with symptoms such as fatigue, weakness, and dizziness [113].

Dyspareunia is another symptom that may be associated with adenomyosis. This pain is often related to painful contractions of the uterus and often indicates the concomitant presence of deep retro cervical endometriosis [80]. Patients with adenomyosis may report deep pain during penetration, particularly when the uterus is manipulated [114].

However, there are also fewer common symptoms that may be observed in some cases, especially the more severe or advanced cases of the disease [116].

Some women with adenomyosis may experience gastrointestinal symptoms such as abdominal pain or intestinal cramping, especially during or just before the menstrual cycle, diarrhea or constipation, which may be influenced by hormonal fluctuations related to the menstrual cycle, and in rare cases rectal bleeding if the adenomyosis involves the lower part of the uterus near the intestine. Adenomyosis can sometimes cause urinary disorders, although these are less frequent than other symptoms. These include urinary urgency, dysuria, and hematuria in rare cases. Chronic fatigue is a less specific symptom but reported by some patients with adenomyosis, especially in the presence of profuse bleeding and secondary anemia [113]. Although pelvic pain is the main symptom, some women with adenomyosis report low back pain that may be associated with uterine enlargement or its anatomical position. Lower back pain may be present during or just before menstruation. In rare cases, women with adenomyosis may present with spotting or non-menstrual vaginal bleeding, occurring between menstrual cycles or during ovulation [114]. It should not be forgotten that some women with adenomyosis may experience psychological or psychosomatic symptoms such as anxiety or depression, and even irritability and emotional stress, due to the chronicity of the pain and discomfort associated with the disease, affecting their quality of life [115].

Several studies have demonstrated that persistent or recurring symptoms like dysmenorrhea and menorrhagia, commonly seen in patients with adenomyosis, tend to have a long-term negative impact on both their physical and mental health, in addition to diminishing their overall quality of life [117]. Patients with adenomyosis who were employed showed a higher likelihood of experiencing anxiety symptoms compared to those who were unemployed. This can be attributed to the substantial effect of adenomyosis on work performance, including increased absenteeism, reduced overall productivity, and greater difficulty in managing daily tasks [118]. Dysmenorrhea is one of the leading causes of recurrent absenteeism among adolescents [119]. A recent systematic review highlighted the significant academic impact of this condition: approximately 20.1% of adolescents with dysmenorrhea report missing school or university due to pain, while 40.9% report a decline in their performance and concentration in class [120]. Other studies have shown that, during exam periods, dysmenorrhea worsens in about half of the cases, contributing to an increased number of missed exams [121,122]. Additionally, adolescents from lower socioeconomic backgrounds tend to have higher rates of school absenteeism compared to those from wealthier families [123]. Iron-deficiency anemia, a common complication in patients with menorrhagic cycles, has been associated with increased levels of psychological stress [124,125]. Furthermore, receiving an infertility diagnosis is recognized as a significant risk factor for developing depression in individuals undergoing assisted reproductive treatments [126,127].

### 5.4. Correlation Between Different Features and Grade of Adenomyosis and Symptoms

Adenomyotic lesions are classified into distinct grades based on the depth of invasion of the myometrium. Lesions can affect different areas of the myometrium, such as the inner myometrium, the outer myometrium, and the junctional zone. The distribution and location of these lesions influence clinical symptoms (Table 2). The classification of degrees of adenomyosis is based primarily on the depth of penetration of the endometrial tissue into the myometrium [128]. Superficial adenomyosis can be identified where lesions involve less than one-third of the thickness of the myometrium. Generally, symptoms are milder, but menstrual pain and heavy menstruation may still be present [115]. At this stage, myometrial involvement is usually limited, with endometrial tissue infiltrating only the inner part of the myometrium, causing local inflammation and mild distortion of the uterine structure [129]. Endometrial tissue may, however, invade up to half the thickness of the myometrium. In this case, the condition becomes more symptomatic, with increased menstrual pain and increased menstrual bleeding [114]. Deep adenomyosis occurs when lesions extend beyond half the thickness of the myometrium and can reach the outer part of the myometrium. This grade is generally associated with more severe symptoms, such as chronic pelvic pain, severe dysmenorrhea, menorrhagia, and, in some cases, infertility. Lesions may eventually involve the entire uterine wall and extend into the surrounding structures [113], becoming advanced adenomyosis. At this stage, the lesions completely penetrate the uterine wall and may even reach the serous layers. It is the rarest and most severe form of adenomyosis and results in significant impairment of quality of life due to persistent and debilitating symptoms [130]. Of particular note is the junctional zone (JZ): Infiltration of this zone contributes to symptoms such as menstrual cramps, profound dyspareunia, and intermenstrual bleeding. It can also increase the risk of infertility by interfering with embryo implantation [129].

In women with adenomyosis, infertility may be caused primarily by local inflammation of the endometrium, especially when lesions penetrate the inner myometrium. The formation of an adenomyosis focus results in platelet aggregation and a hypoxic condition, which stimulates the production of inflammatory cytokines, prostaglandins, and an increase in local estrogen synthesis [131]. These processes can cause uterine hyperperistalsis, mediated by the activation of the estrogen receptor, which induces oxytocin signaling, as well as promoting fibrosis through the epithelial–mesenchymal transition process and the transformation of fibroblasts into myofibroblasts. Altered contractility of the JZE may result from hypoestrogenism, causing a vicious cycle of hyperperistalsis and autotraumatisation [132]. Although abnormal contractions are believed to disrupt gamete and embryo transport, local inflammation and fibrosis are considered determinants of an altered uterine environment that is not conducive to embryo implantation. In the endometrium of affected women, a reduction in microvilli, a change in steroid hormone metabolism, and an increase in oxidative stress are observed [103,109].

Furthermore, in affected patients, the endometrium of the junctional zone appears thickened and disrupted. Adenomyosis does indeed appear to be associated with reduced pregnancy rates, fewer live births, more miscarriages, and unfavorable obstetric pregnancy and neonatal outcomes [103,133].

The pathogenic mechanisms behind these complications are multiple, for example, incomplete or absent remodeling of the spiral arteries during pregnancy within the JZE, resulting in uterine peristalsis during the luteal phase. Abnormal uterine contractility is associated with non-progression, uterine hyperstimulation and atony, placental retention, and postpartum hemorrhage; moreover, in addition to increased intrauterine oxidative stress that may lead to maternal endothelial dysfunction underlying abnormal placentation, the spiral arteries may undergo hyperplastic changes, resulting in increased flow impedance of the uterine arteries and placentation defect.

In addition, women with adenomyosis appear to have a significantly higher risk of developing preeclampsia and delivering a small for gestational age (SGA) infant. Again, an association between these two outcomes is likely, biologically plausible, and consistent with the mechanisms of poor placentation and endothelial inflammation [134].

Nirgianakis et al. [135] provided a more detailed analysis of pregnancy, obstetric, and neonatal outcomes.

It is possible to identify a statistically significant risk for preterm delivery (<37 weeks) but not for severe preterm delivery (<32 weeks). Regarding gestational hypertension, the only study with this result available [136] showed an increased risk in the adenomyosis group. Regarding postpartum hemorrhage, the meta-analysis showed a significantly higher risk in the adenomyosis group.

In patients with adenomyosis, there is also a statistically significant risk of intrauterine growth restriction (IUGR) in fetuses, with possibly related unfavorable neonatal outcomes.

The mechanisms through which adenomyosis may increase the risk of complications involve several factors. Chronic inflammation, which leads to local persistent inflammation, can damage blood vessels and disrupt endothelial function, thereby raising the likelihood of conditions such as preeclampsia and gestational hypertension. Additionally, altered uterine and placental vascularization may impair placental blood flow, reducing the supply of nutrients and oxygen to the fetus, which can result in conditions like SGA/IUGR. Furthermore, adenomyosis can cause immune system dysregulation, disrupting the normal regulation of the placenta and contributing to complications such as preeclampsia and other pregnancy-related disorders. A comprehensive understanding of these mechanisms is crucial for the optimal management of pregnancies in women with adenomyosis, as early diagnosis and careful monitoring can help prevent or reduce the risk of complications for both the mother and the fetus.

## 6. Diagnosis

### 6.1. Endometriosis

The diagnosis of endometriosis is a clinical challenge due to its heterogeneous presentation and variable symptomatology. Initial suspicion typically arises from clinical history, including symptoms like dysmenorrhea, deep dyspareunia, dysuria, dyschezia, painful rectal bleeding, hematuria, or infertility. A thorough pelvic examination can provide further indications, particularly when deep nodules in the anterior or posterior compartments or endometriomas are present. However, for superficial peritoneal endometriosis, the clinical exam may not reveal abnormalities [137,138].

Imaging plays a critical role in the diagnostic pathway. TVUS is considered the first-line imaging modality, while MRI serves as a second-line option to further evaluate suspected cases. Both techniques have demonstrated high sensitivity and specificity for DIE. However, the absence of imaging findings does not exclude endometriosis, particularly in cases of superficial peritoneal disease. In such cases, diagnostic laparoscopy or empirical medical management with hormonal treatments can be considered [1,139,140].

Historically, a definitive diagnosis of endometriosis required histological confirmation via laparoscopic biopsy. Although advances in imaging now permit non-invasive diagnosis in many cases, laparoscopy is still warranted for women with unresolved symptoms or when imaging is inconclusive, as it allows for the direct visualization and biopsy of endometriotic lesions.

TVUS remains the most practical and widely used method for preoperative evaluation, while MRI may be employed in complex cases for further characterization. Tumor marker CA-125 lacks specificity for diagnosing endometriosis or early-stage endometriosis-associated ovarian cancer (EAOC), as its moderate elevations are also seen in benign conditions [141,142,143,144].

When evaluating ovarian endometriomas, typical ultrasound features include a unilocular or minimally multilocular cyst with a homogenous “ground-glass” appearance and no solid or vascularized papillary structures. In contrast, the presence of vascularized solid components may indicate a higher risk of borderline or malignant transformation. Risk factors for ovarian cancer in women with endometriomas include age ≥ 45 years at first diagnosis and endometriomas > 8 cm [145]. For complex or unclear ovarian findings, MRI with contrast can be useful to differentiate benign from malignant lesions. If a neoplastic process is suspected, a comprehensive CT scan should be conducted [141,145,146].

Endometriosis should also be considered in patients presenting with symptoms linked to uncommon locations, such as the diaphragm, lungs, or surgical scars, where cyclical chest pain, hemoptysis, shoulder pain, or scar swelling may indicate ectopic endometriotic implants [1].

Ultimately, a comprehensive approach combining clinical assessment, imaging, and, when necessary, surgical intervention remains the gold standard for accurate diagnosis and individualized management of endometriosis.

### 6.2. Adenomyosis

The diagnosis of adenomyosis has evolved significantly over time, shifting from reliance on histopathological confirmation to non-invasive imaging techniques. While histological confirmation was once the gold standard, today’s approach primarily involves imaging modalities that provide accurate diagnosis and guide treatment strategies. TVUS is typically the first-line diagnostic tool due to its accessibility and cost-effectiveness, while MRI serves as a second-line modality for cases requiring further evaluation [147].

TVUS is valuable for identifying typical adenomyotic features, including a thickened junctional zone, myometrial cysts, asymmetrical myometrial thickening, and heterogeneous myometrial echotexture. The Morphological Uterus Sonographic Assessment (MUSA) criteria have standardized the evaluation of these findings by categorizing them into direct and indirect features [148,149]. Nevertheless, it is important to underline that medical therapy can change the US features, both direct and indirect signs [150].

Direct signs indicate the presence of ectopic endometrial tissue, such as myometrial cysts or hyperechogenic islands, while indirect signs include an enlarged, globular uterus or a disrupted junctional zone [151,152].

The number of these features is not necessarily proportional to the severity of the disease, as extensive adenomyosis can present with few visible signs, while focal forms may display multiple features [153].

MRI, with its superior tissue characterization, is often utilized when ultrasound findings are inconclusive or when a more comprehensive assessment is necessary [154].

Typical MRI findings include a thickened junctional zone (≥12 mm), poorly defined borders between the endometrium and myometrium, and low signal intensity on T2-weighted images, indicating myometrial hypertrophy and ectopic endometrial tissue [155,156,157,158].

However, junctional zone thickness can vary based on factors like menstrual cycle phase and hormonal treatment, which may complicate interpretation [151,154,159].

Additional diagnostic techniques, such as sonohysterography and hysteroscopy, can complement imaging in specific scenarios. Sonohysterography, through the infusion of saline into the uterine cavity, can reveal subendometrial cystic spaces characteristic of adenomyosis, while hysteroscopy can identify endometrial defects and hypervascularization suggestive of the condition. Although helpful, these techniques are not definitive for diagnosis and should be used in conjunction with imaging [160].

Emerging technologies, like elastography, are being explored to differentiate adenomyosis from other myometrial pathologies, such as fibroids. By measuring tissue stiffness, elastography may be used to enhance the accuracy of TVUS, yet its diagnostic utility requires further validation [161,162]. Thus, while non-invasive methods have improved diagnostic precision, the standardization of criteria and comprehensive classification systems is still needed to ensure consistency and accuracy in clinical practice.

## 7. Progression of Disease

### 7.1. Endometriosis

The progression of endometriosis is driven by a multifactorial interplay of hormonal dysregulation, immune dysfunction, and genetic predisposition, leading to the persistent growth and infiltration of ectopic endometrial tissue into adjacent structures. Clinical observations and longitudinal studies indicate that, in early-stage disease, approximately one-third of cases remain stable, one-third regress, and another third show progression if left untreated. Specifically, deep endometriosis (DE) has been reported to progress in just over one-third of affected individuals. In contrast, most women diagnosed via ultrasound with ovarian endometriomas exhibit minimal or no significant increase in cyst size over time, suggesting that a conservative, expectant management approach may be appropriate in many cases [163,164].

Several theories exist to explain its development, such as retrograde menstruation, coelomic metaplasia, genetic predisposition, immune dysregulation, and environmental factors. The disease typically follows a sequence of recurrent bleeding, inflammation, fibrin deposition, adhesion formation, and scarring, resulting in pelvic anatomical distortions and increased symptom severity, such as chronic pelvic pain and organ dysfunction.

The natural course of endometriosis varies widely among individuals; some may experience slow, limited progression, while others develop rapidly worsening disease with extensive pelvic involvement. Factors such as early age at onset, high local estrogen levels, and coexisting conditions can accelerate disease progression. Additionally, the deep infiltration of lesions often compromises the function of pelvic organs, particularly the bowel, bladder, and uterosacral ligaments, complicating surgical management and increasing recurrence risk, even after complete resection [165,166,167].

One of the major challenges in managing endometriosis is its recurrent nature. Even with medical or surgical treatment, residual lesions or incomplete excision create a chronic inflammatory environment that promotes angiogenesis and nerve fiber formation within lesions, further embedding the disease and contributing to persistent symptoms.

Although endometriosis is generally benign, OMAs can rarely undergo malignant transformation into EAOC. The most common histotypes include clear cell carcinoma (CCC) and endometrioid ovarian carcinoma (EnOC), both classified as type I ovarian tumors. The pathogenesis of malignant transformation is still under investigation; however, recurrent hemorrhage within OMAs is thought to generate a pro-oxidative environment, leading to DNA damage and genomic instability. According to a second possible mechanism, endometriotic cells adapt to oxidative stress with the help of macrophages, which promote antioxidative defenses and reshape the cellular environment, thereby facilitating malignant transformation. This, coupled with mutations in key genes like PIK3CA, ARID1A, and PTEN, as well as microsatellite instability, can promote oncogenic pathways, highlighting the need for vigilant monitoring and individualized management, particularly in patients with extensive or long-standing endometriosis [165,168].

Risk factors for malignant transformation include older age at diagnosis, large endometriomas (≥8 cm), solid components within the cyst, postmenopausal status, nulliparity, and elevated estrogen levels. EAOCs tend to manifest in women aged 35–55, and early detection is challenging as carcinomas may initially arise from small, atypical epithelial areas within an endometrioma, making them difficult to distinguish from benign lesions using standard imaging techniques. This highlights the need for careful monitoring and individualized management in high-risk patients [169,170].

### 7.2. Adenomyosis

Adenomyosis progression is influenced by hormonal imbalances, inflammatory responses, and local tissue remodeling. Its development is typically slow and varies significantly between individuals, depending on factors such as hormonal environment, parity, and coexisting gynecological conditions. Initially, adenomyotic lesions may appear as localized foci within the myometrium; however, over time, they can expand, becoming more diffuse and involving a greater portion of the uterine wall, resulting in increased uterine volume and cavity deformation [171].

The condition’s progression is influenced by repeated cycles of bleeding and chronic inflammation within the ectopic endometrial tissue, which stimulate myometrial hyperplasia and fibrosis, resulting in thickening of the uterine wall, increased uterine volume, and the development of nodular or cystic formations within the myometrium. The interaction between endometrial and myometrial components further remodels the uterine structure, contributing to symptoms like heavy menstrual bleeding, dysmenorrhea, and chronic pelvic pain.

Key mechanisms include increased expression of matrix metalloproteinases (MMPs) and altered extracellular matrix components, weakening the junctional zone (JZ) between the endometrium and myometrium. Chronic inflammation, driven by elevated levels of pro-inflammatory cytokines, such as interleukin-6 (IL-6) and TNF-α, perpetuates tissue injury and fibrosis. Additionally, hormonal dysregulation, particularly increased local estrogen production due to elevated aromatase activity in adenomyotic lesions, promotes the proliferation of ectopic endometrial cells and induces resistance to apoptosis. This enhanced estrogenic environment also contributes to the overexpression of PR isoforms, which, paradoxically, leads to progesterone resistance, considered a hallmark of the disease, sustaining the growth and persistence of adenomyotic tissue. As adenomyosis progresses, the distorted myometrial structure disrupts uterine contractility, causing abnormal uterine bleeding and impacting fertility [172]. The distorted uterine anatomy may interfere with normal sperm transport, embryo implantation, and pregnancy maintenance, thereby increasing the risk of miscarriage and adverse pregnancy outcomes. In severe cases, adenomyosis may coexist with other uterine pathologies, such as leiomyomas or endometriosis, complicating its clinical presentation and management [173,174,175].

Although adenomyosis is generally considered a benign condition, its progressive nature can significantly affect quality of life and reproductive outcomes. Understanding the mechanisms of disease progression is crucial for optimizing targeted therapeutic approaches.

## 8. Follow Up

The management of endometriosis and adenomyosis primarily aims to manage pain, improve quality of life, and preserve fertility, tailored to each patient’s symptoms and reproductive goals [1]. While we have recommendations for different medical and surgical treatments, there is no guideline recommending adequate follow-up times after diagnosis of these diseases [176].

Therefore, the role of clinical and ultrasonographic follow-up at 6 months in the first year of the intake, and every 6 to 12 months thereafter, appears to be important not only in assessing the new appearance of signs of disease, but also in helping the patient to have better therapy compliance and quality of life [88].

In fact, it is essential to evaluate the patient’s response to therapy over time and adapt it to the individual patient. Hypoestrogenism must be managed differently depending on the patient’s age, BMI, and the appearance of side effects.

In adolescents and young girls, after 6 months of continuous progestin therapy, it may be useful to switch to a continuous estrogen–progestin to reduce the effects of hypoestrogenism.

When using estrogen–progestins, it is also important to choose the dosage of the estrogenic component based on the patient’s age and BMI. While high-dose estrogens may be useful in young girls with a normal or reduced BMI, low-dose and/or natural estrogens may be useful in older women or with a high BMI.

**Figure 2:**
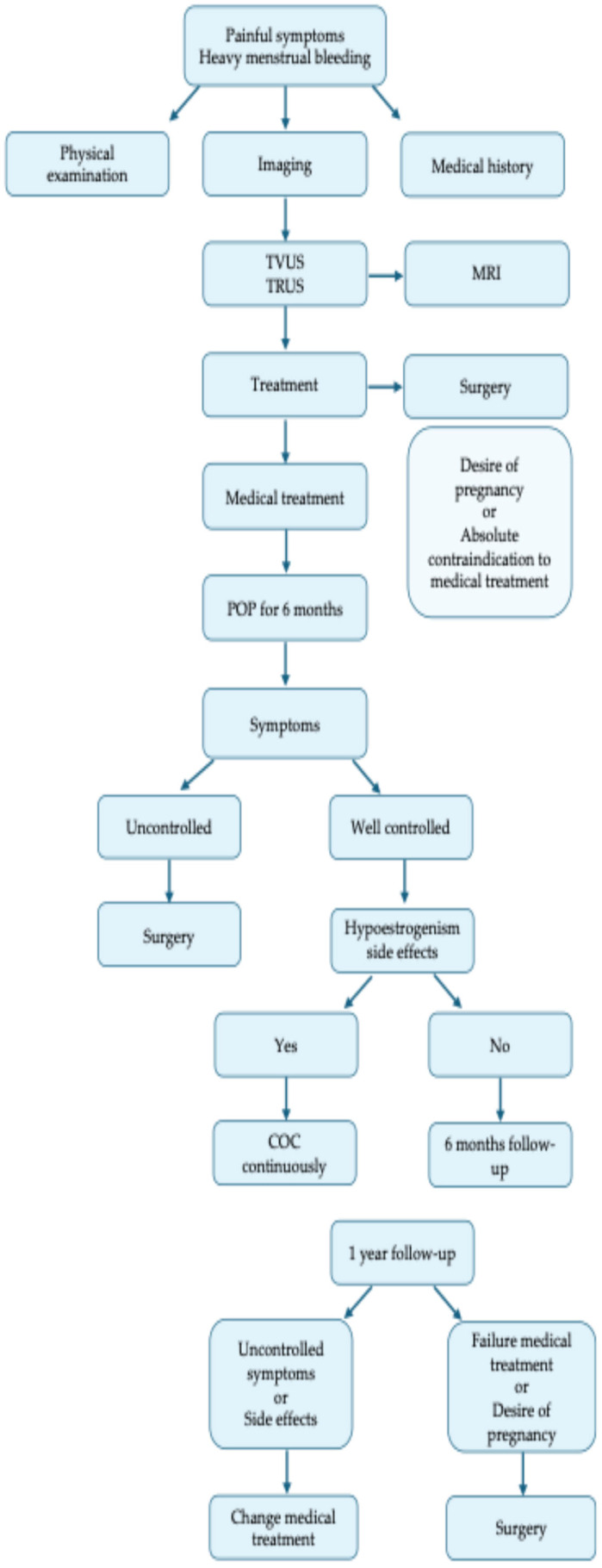
Diagram of management.

Furthermore, patients with signs of endometriosis also have a benefit in follow-up to monitor the progress of the disease over time [177,178,179] and possibly modify the medical or surgical treatment approach to evaluate the change of medical therapy or the surgical approach (Figure 2).

## 9. Conclusions

Endometriosis and adenomyosis are chronic hormone-related pathologies. It is essential to make an early diagnosis for adequate therapeutic management, in which medical hormone therapy is the first line of treatment. To maximize compliance with therapy, these patients should be followed over time, and hormone therapy should be adapted to individual patients. Other than improving the patients’ quality of life, the medical therapy appears to reduce disease progression extent and degree. The long-term follow-up of patients allows for monitoring treatment adherence, leading to improved outcomes. Furthermore, establishing a continuous, trust-based relationship with patients enables modifications to treatment in response to changes in patient needs, whether due to complications, symptoms, or a desire for pregnancy. The impact on patients’ personal lives is also evident, as they are significantly affected by the presence of disabling and wide-ranging symptoms. Therefore, prioritizing the mental health of individuals with chronic conditions could substantially improve their quality of life and significantly enhance the effectiveness of medical treatments.

## Figures and Tables

**Table 1:** Endometriosis and symptoms.

Features of Endometriosis	Symptoms
OMA	Dysmenorrhea, no specific symptoms
DIE (USL; RVS)	Dysmenorrhea, dyspareunia
Bowel	Dysmenorrhea, dyschezia, functional intestinal symptoms
UTE (Bladder, ureter)	Dysmenorrhea, dysuria, flank pain
Adhesions	Dysmenorrhea, chronic pelvic pain

OMA: Endometrioma, DIE: Deep Infiltrating Endometriosis, USL: Uterosacral Ligaments, RSV: Recto-Vaginal Septum, UTE: Urinary tract endometriosis.

**Table 2:** Adenomyosis and symptoms.

Type of Adenomyosis	Symptoms
Diffuse	Dysmenorrhea, HMB
Adenomyoma	Dysmenorrhea
Focal	Dysmenorrhea
**Myometrial involvement**	
Internal	Dysmenorrhea, RPL
External	HMB, pregnancy complications

HMB: Heavy Menstrual Bleeding, RPL: Recurrent Pregnancy Lost.

## Data Availability

Not applicable.
